# Arousal regulation and affective adaptation to human responsiveness by a robot that explores and learns a novel environment

**DOI:** 10.3389/fnbot.2014.00017

**Published:** 2014-05-14

**Authors:** Antoine Hiolle, Matthew Lewis, Lola Cañamero

**Affiliations:** Embodied Emotion, Cognition, and (Inter-)Action Lab, School of Computer Science and STRI, University of HertfordshireHatfield, UK

**Keywords:** developmental robotics, social robotics, affective adaptation, human–robot interaction, arousal, attachment bonds, emotion regulation, autonomous robots

## Abstract

In the context of our work in developmental robotics regarding robot–human caregiver interactions, in this paper we investigate how a “baby” robot that explores and learns novel environments can adapt its affective regulatory behavior of soliciting help from a “caregiver” to the preferences shown by the caregiver in terms of varying responsiveness. We build on two strands of previous work that assessed independently (a) the differences between two “idealized” robot profiles—a “needy” and an “independent” robot—in terms of their use of a caregiver as a means to regulate the “stress” (arousal) produced by the exploration and learning of a novel environment, and (b) the effects on the robot behaviors of two caregiving profiles varying in their responsiveness—“responsive” and “non-responsive”—to the regulatory requests of the robot. Going beyond previous work, in this paper we (a) assess the effects that the varying regulatory behavior of the two robot profiles has on the exploratory and learning patterns of the robots; (b) bring together the two strands previously investigated in isolation and take a step further by endowing the robot with the capability to *adapt* its regulatory behavior along the “needy” and “independent” axis as a function of the varying responsiveness of the caregiver; and (c) analyze the effects that the varying regulatory behavior has on the exploratory and learning patterns of the adaptive robot.

## 1. Introduction

For autonomous robots having to operate in human real-world environments, adaptation to incompletely known and changing environments and personalization to their human users are key features for successful integration in such environments, which includes the ability to sustain long-term interactions with the humans. Adaptation involves autonomous exploration and learning but, as argued elsewhere (Cañamero et al., 2006), excessive autonomy and pro-activeness on the part of the robot might be negative since, among other things, it might carry risks of robots not being appropriately adapted to humans, behaving “selfishly” and being uncaring and detached of their human users. A balanced combination of autonomy, pro-activeness and user-driven interaction is thus needed for robots to operate in human environments and interact with humans as and when it is better suited to the humans. Such a combination is highly variable, differing across individuals, concrete situations, cultures, etc. For example, a person might prefer to teach the robot or guide their learning explicitly on some occasions, whereas at other times the same person might not be available or might prefer not to be bothered by the robot and will let it try to solve on its own the problems that it encounters. It is thus important that the robot can adapt to the preferences of the human at different points in time.

For smooth interaction, it is also important to simplify the task of the human in terms of (a) understanding when the robot needs help and (b) how to signal to the robot when s/he is willing to help and when s/he prefers not to interact. A natural way for the robot to convey to the human its need for help is to directly solicit help. A natural way for the human to show the robot when s/he is willing to help is by attending or ignoring the robot's request. In this paper we propose to use these natural interaction signals for the robot to adapt to the interaction preferences of the human at different points in time, and vary between a more “needy” (soliciting more help) or a more “independent” (soliciting less help) interaction profile as a function of the varying responsiveness of the human present in the environment.

In particular, in the context of our work in the area of developmental robotics regarding robot-caregiver interactions, in this paper we investigate how a “baby” robot that explores and learns novel environments can adapt its affective regulatory behavior of soliciting help from a human “caregiver” to the preferences shown by the human in terms of varying responsiveness.

The varying responsiveness of the caregiver and its effect on the affective regulatory behavior of the robot has in turn an effect on how the robot explores and learns its environment. In previous work regarding the differences in regulatory behaviors used by “needy” and “independent” robots to cope with the “anxiety” (arousal) arising from novelty in the environment, we incidentally observed that, due to natural interaction dynamics, the exploration and learning patterns of both robots were also different. The fact that the adaptive robot architecture presented in this paper can now vary between a “needy” and an “independent” interaction profile to adapt to the responsiveness of the human, means that variations in the responsiveness of the human also affect the exploration and learning patterns of the robot, in addition to changes in the regulatory behaviors used. This is an important feature from the perspective of human–robot interaction as it opens up the door to the possibility that, by intentionally varying his/her responsiveness, the human could intentionally bias the exploration and learning of the robot toward learning aspects of the environment that are useful for the human.

We thus present a robot architecture and three sets of experiments assessing the interplay between affective variables—namely the level of arousal of the robot as a function of the novelty and complexity of the environment, and the comfort provided by a caregiver to help regulate that arousal—in dyadic robot-(human) caregiver interactions, and their effects on the regulatory, exploratory, and learning behaviors of the robot. The robot architecture and the dynamics of the model take inspiration from psychological and neurobiological theories and findings regarding the development of attachment bonds on mother-infant interactions in the early years of life.

The principles underlying the model used are the following. An autonomous robot explores the environment and tries to learn and categorize its perceptual features. In real time, the architecture evaluates to which extent the current features are novel or already learned, following Berlyne's notion of “collative variables” (Berlyne, 1954, 1960), which are stimulus properties such as complexity, novelty, surprisingness, or incongruity. Berlyne hypothesized that the perception of stimuli high in these properties results in the arousal of internal conflicting responses in the organism, and the organism continues interacting with the stimuli until the conflict has been resolved. Following this hypothesis, and in our architecture, low arousal levels trigger exploration, whereas higher and sustained levels have the robot continue interacting with the stimulus and trigger regulatory behaviors that can help resolve the conflict; in the first years of life (and in our model) such regulatory behaviors are often aimed at attracting the attention and help of a caregiver. In our model, collative variables are used to measure an arousal level which is correlated with the novelty or incongruence of the current perceptions. If the current perceptions are known, the measures will lead to a low arousal level, and the robot will explore further. If these measures and the arousal are at a medium level, the robot continues attending the current stimuli. If the arousal is high, the situation is akin to a “stressful” episode and the robot will trigger a regulatory behavior to request the attention of a human present in the environment, as infants do in stressful situations. Distal or proximal intervention from the human “caregiver” provides comfort to the robot, which in turn reduces the arousal level of the robot, leading to further exploration and learning. Prolonged lack of feedback from the caregiver leads to a different regulatory behavior akin to slow self-regulation of arousal, which also ends up leading to further exploration and learning but with a different dynamics.

### 1.1. Research questions

The research presented in this paper takes inspiration from attachment theory and its findings, which we use as a model to bootstrap the (cognitive, social and emotional) development of autonomous robots in interaction with humans. The literature on attachment theory is very large and models vary. To provide the principal background to our model stemming from psychology (which we will develop in more detail in section 1.2), we summarize here the main principles that provide our conceptual framework regarding the attachment subsystem and its interplay with learning and development:

1. The (primary) caregiver or “attachment figure” provides a “secure base” from which an infant can venture out to explore the world safely and to which s/he can return in case of need or distress.
2. The bond between an infant and his/her attachment figure is mainly characterized by the calming influence that the attachment figure has on the infant in times of distress, confusion, or fear.
3. This influence is mediated through social interactions between the dyad, which can be distal (e.g., being visible at a “comfortable” distance), or proximal (through physical interaction such as touching and patting).
4. Different types of interaction and “caregiving profiles” affect differentially the cognitive and affective development of infants.
5. Whereas the resulting attachment bond can have different quality (typically ranging from “secure” to “insecure”), there is no universal “golden standard” regarding a caregiving style to achieve an attachment bond of high quality. Different styles can be more or less suited to different characteristics of the infant (e.g., in terms of strategies used to regulate stress) and vice versa, and are strongly influenced by society and culture.

The substantial developmental and comparative psychology literature on the subject (e.g., Ainsworth and Bell, 1970; Ainsworth et al., 1978; Cassidy and Shaver, 2008; van Ijzendoorn et al., 2009; Bard et al., 2014) has mostly studied attachment bonds in the context of the “negative episodes” (distress, confusion, or fear) experienced by infants of around 1 year of age elicited by the introduction of an element external to the dyad, typically called a “stranger.” This type of stressful situations was used to elaborate what has become the paradigmatic standard test to assess the “quality” of the attachment bond between an infant and his/her primary carer—the Strange Situation Procedure (Ainsworth and Bell, 1970; Ainsworth et al., 1978), cf. section 1.2.1.

Although not as extensively studied by attachment theorists, learning and exploration episodes are also potentially stressful due to the novelty of the environment and the complexity of the objects and agents the infant can interact with. Our work has being focusing in this aspect (Blanchard and Cañamero, 2005, 2006; Hiolle and Cañamero, 2008; Hiolle et al., 2012; Lones et al., 2013) since (increasingly) autonomous and safe exploration and learning of novel environments is a crucial skill that autonomous robots must develop. Robots inhabiting human environments could beneficially develop this skill in interaction with humans (Cañamero et al., 2006). In our work we have also investigated the research question of how a human can positively influence the exploration patterns and learning outcomes of a developing robot endowed with an attachment subsystem. This attachment subsystem is responsible for triggering requests for help or assistance based on the robot's assessment of the current situation and its novelty or complexity (leading to distress), reflecting the ability of the robot to learn and assimilate the current features of the environment. Mediated by an evaluation of comfort provided, the human caregiver can alleviate the distress of the robot and bias its assessment of the current situation. In turn, the interaction between the robot's assessment of the features of the environments and the social interactions with the human shapes exploratory behavior, and therefore what and how the robot learns as a function of the behavior of the human.

Our previous work has investigated issues related to principles 1–4 from the above list. The study presented in this paper is related to principles 4 and 5 and their interplay. In this context, we investigate specifically how a robot that explores and learns novel environments can adapt its affective regulatory behavior to that of a human “caregiver” as a function of the varying responsiveness of the caregiver. In particular, we set out to research:

(Q1) Whether and how different caregiving styles can be more or less suited to different characteristics of the “infant” (robot) in terms of strategies used to regulate stress.
(Q2) Whether and how different types of interaction and “caregiving profiles” might affect differentially the cognitive and affective development of the “infant” (robot)—namely its regulatory, exploratory and learning patterns.
(Q3) The use of adaptation as a mechanism to produce a suitable match between different robot profiles and caregiving styles.

We designed three experiments to investigate these three questions.

### 1.2. Theoretical background

Since the pioneering work of John Bowlby regarding the role and the dynamics of attachment behaviors in development of infants from an early age (Bowlby, 1958, 1969), much research work in Developmental and Comparative Psychology has been devoted to address the impact of attachment dynamics on the socio-cognitive and emotional development of infants, both in human and non-human primates (see e.g., Cassidy and Shaver, 2008; van Ijzendoorn et al., 2009; Bard et al., 2014).

As Bowlby highlighted following his observations of behaviors and affective displays in infants, a primary attachment figure—often the mother—plays a central role in regulating and orienting the infant during stressful periods, and during play- and learning-oriented interactions. Bowlby developed a control systems theory of this aspect of social interaction, where proximity-seeking regulatory behaviors are produced by the infant as a response to the distress felt. These proximity-seeking behaviors serve to attract and maintain the attention of the caregiver, and also help to regulate the developing emotions of the infant (Sroufe and Waters, 1977).

#### 1.2.1. The quality of attachment bonds

The strategies employed by infants to attract and maintain the attention of the caregiver and for emotion regulation are different in their nature and time line, and Ainsworth endeavored to categorize them in patterns of attachment with a standardized test (Ainsworth and Bell, 1970; Ainsworth et al., 1978). This test, called the *Strange Situation Procedure*, confronts the infant to a stressful event—being left alone with an unknown adult or “stranger”—and assesses the responses of the infant when s/he is reunited with the caregiver in terms of categories corresponding to qualitatively different types of attachment profiles. The proposed categories draw a clear distinction between “securely” and “insecurely” attached children, with the latter further divided into subcategories “ambivalent” and “avoidant,” and a fourth category “other” for cases that do not clearly show one of the above profiles. Securely attached children are able to cope with periods of distress, and “trust” their primary caregiver in their ability to return and respond to their needs. Insecurely attached children do not respond to their caregivers in a consistent manner, and tend to exhibit longer and more frequent behaviors related to negative affect such as crying and rejecting the approaches of their caregiver.

#### 1.2.2. A secure base for development

Bowlby introduced the notion of *Secure Base* to reflect the role that caregivers play in grounding the exploratory behaviors of infants, qualifying the attachment figure as an affective safe haven from which to explore (Bowlby, 1988; Waters and Cummings, 2000) and develop social, affective and cognitive skills in a successful manner. Since then, much work has been devoted to the study of the factors responsible for the emergence of these patterns of behaviors, and the impact of the behavior of the attachment figure in their development (Waters et al., 1994; Mikulincer et al., 2003; Mills-Koonce et al., 2007; van Ijzendoorn et al., 2009). The notions of *Sensitivity*—the ability to correctly interpret the behavior and demands of the infant—and of *responsiveness*—the timeliness of the responses of the caregiver—have been investigated to account for the secure/insecure difference in attachment behaviors (Bell and Ainsworth, 1972). These factors have been related to presence or absence of physical and emotional availability of a caregiver, and are thought to be determinants of the affect of the infant (Field, 1994).

#### 1.2.3. The neurophysiology of infant–mother interactions

A strong body of evidence suggests that the neurophysiological basis for infant attachment and distress responses would be regulated by the release of endogenous opioids (Nelson and Panksepp, 1998; Gray et al., 2000; Weller and Feldman, 2003). The stress responses of the infant are correlated with increase in cortisol levels (Liu et al., 1997), and in turn the opioid system can reduce these levels. As emphasized in Smith and Stevens (1996); Stevens and Zhang (2009), the regulatory processes involved in this can be modeled as differential equations including the effects of the endogenous opioids in intensity and duration, the responsiveness of the caregiver, and the frequency of the interactions. To summarize, the consensus on the dynamics of the physiological substrates of attachment and mother–infant interactions is that the comfort provided by a caregiver promotes the release of opioids which in turn calm and soothe the infant. The lack of release of these endogenous opioids would trigger what Bowlby designated as attachment system, and therefore the behaviors aimed at regulating this imbalance.

#### 1.2.4. Arousal modulation of dyadic interaction systems

Throughout the years, the notion of *arousal* has been used in psychological theories to measure and quantify states of heightened activity, alertness, and attention, and was originally believed to reflect the activation of part of the central nervous system. This notion lead Hebb to propose a theory of drives based on an arousal system (Hebb, 1955). Moreover, arousal is an often used measure for human subjects to report their emotional state in questionnaires following an experimental manipulation and the modelization of emotion in a two dimensional axis of Arousal and Valence (Russel, 1980). Alongside Hebb's work on the relationship between arousal, drives and goal-oriented behaviors, Berlyne postulated in his theory of curiosity (Berlyne, 1954, 1960) that low levels of arousal trigger exploratory behaviors whereas internal conflicts between expectations and the stimuli perceived give rise to a higher level of arousal. He added that the exploratory behaviors serve to promote a medium-to-optimal level of arousal. Berlyne hypothesized that arousal was a construct relating to “collative variables” and related them to exploratory behaviors as follows:

The probability and direction of specific exploratory responses can apparently be influenced by many properties of external stimulation, as well as by many intraorganism variables. They can, no doubt, be influenced by stimulus intensity, color, pitch, and association with biological gratification and punishment,… [but] the paramount determinants of specific exploration are, however, a group of stimulus properties to which we commonly refer by such words as ‘novelty’, ‘change’, ‘surprisingness’, ‘incongruity’, ‘complexity’, ‘ambiguity’, and ‘indistinctiveness’. (Berlyne, 1965, p. 245).

Furthermore, Berlyne formulated the notion of arousal as “all the stimulus properties that go to make up arousal potential, including the “collative” properties, e.g., novelty, variability, surprisingness, complexity, and ambiguity.” (Berlyne, 1969, p. 1068).

Arousal has also been investigated in terms of optimal functioning during knowledge acquisition and retention. A debate has grown centered on the “Inverted U-Shape hypothesis” (Anderson, 1990; Baldi and Bucherelli, 2005), which posits that physiological and cognitive functions are influenced by the Arousal level in a non-linear manner, and that an optimal medium level exists at which optimality can be attained for memory and physical tasks. With regards to caregiver–infant dyadic studies Feldman (2003) showed that the co-regulation of positive arousal between mother–infant and father–infant displayed cycles between low and medium levels, or high and medium levels, depending on the style and gender of the caregiver. This reinforces the view that infants are subject to these cyclic arousal fluctuations. Moreover, these cycles seem to occur fast and reflect a real-time state of the interaction.

### 1.3. Related work in robotics

#### 1.3.1. Arousal

Arousal has been used in artificial and robotic systems for different purposes. It has for example been used as a parameter to control the emotional displays of a robot as a function that reflects the levels of external stimulation received by an agent (Breazeal and Scassellati, 1999; Breazeal, 2003). Ogino et al. (2013) propose a motivational model of early parent–infant communication. Their model is based on the need for relatedness and its relationship to the dynamics of the pleasure and arousal in face-to-face interactions. They tested their architecture using a virtual robot on a computer which interacted with a human playing the role of the parent. To that end, their model includes a two-dimensional vector of pleasure and arousal following the circumplex model of emotions introduced by Russel (1980). The arousal of the agent is computed with respect to measures of novelty, stress and the perceived arousal of the human. The pleasure varies proportionally to the pleasure perceived, the relatedness, and the expectancy of the perception of some emotion in the human. Their study intended to reproduce the phenomenology observed during mother–infant interactions and especially during still face episodes (Tronick et al., 1979; Adamson and Frick, 2003; Nadel et al., 2005). These episodes are characterized by a decrease in pleasure and positive emotions when the attachment figure stops responding to the infant's positive signals, such as gazing and smiling. The results they present show that this model reproduces the typical drop in positive affect following a still-face episode. Although the architecture based its novelty on a predictive system learning the likeliest next action the caregiver would produce, the interplay between the behavior of the caregiver and the exploratory behavior and learning of the robot were not studied.

#### 1.3.2. The attachment system

In the few studies trying to model the attachment system and its dynamics, the behaviors related to attachment and their occurrence are studied in isolation from other important facets of (infant) development. Typically, the socio-cognitive development is left aside, the attachment subsystem is considered on its own, and the analysis is solely concerned with the success or failure of a coping strategy or a regulatory behavior. For instance, Petters (2006a) presents simulations of caregiver–infant interactions using several control architectures based on attachment theory. The main goal of these simulations of artificial agents interactions was to model the relationships between the goals and behaviors observed in young infants. The resulting architectures were tested in unsafe or safe (secure or insecure) scenarios. Depending on parameters relating to the sensitivity of the caregiver of the infant agent, the behavior of the infant would vary. Specifically, the architectures comprised several main components inspired by the literature on Attachment theory. First, an Anxiety internal variable increases when the perceptual appraisal of the situation was deemed unfamiliar or unsafe. A Warmth internal variable was introduced to evaluate the positive interactions with the caregiver as hypothesized in the Secure Base paradigm. Based on these internal variables and the current perceptions, the action selection system assigns weights to the current goals and a winner-take-all approach is used to trigger the behavior associated with the most active one. Several variations of this architecture have been tested to include learning and adaption from previous interactions. This adaptation was based on the success or failure to regulate the internal variables, with similar dynamics to the Animat approach to motivational systems (Cañamero, 1997; Avila-Garcia and Cañamero, 2004; Cañamero and Avila-García, 2007). For instance, the agent tries to approach its caregiver when the Anxiety variable is high, and the responsiveness or sensitivity of the carer (a built-in constant in the simulation) defines if the carer will provide Warmth and relieve the Anxiety. The reported results clearly show some emergent categories which are believed to correspond to the ones Ainsworth brought to light (Ainsworth et al., 1978). However, the attachment behavior itself is considered aside from the exploration and its potential consequences on development.

In contrast with the models developed and tested in simulations in various studies concerning the emergence of attachment patterns (Smith and Stevens, 2002; Petters, 2006b; Stevens and Zhang, 2009), we have studied the dynamics of the dyadic interactions in a robot-centric manner. Our main aim is to improve the adaptivity of autonomous robots in order to, on the one hand, support their autonomous learning as a function of its interactions with the physical and social environment, and on the other hand improve the affective experience of the human in human–robot dyadic social interactions.

However, despite the differences with the other body of work that models attachment dynamics and arousal modulation, we share the common view of the basic interplay between caring styles and behavior variations in affective adaptation. Indeed, these simulation models attempt to have a specific pattern of behavior emerge across several interactions based on a stereotypical caregiving style. This style is based on a *sensitivity* and *responsiveness* formalism, similarly to our work.

#### 1.3.3. Exploration, curiosity, and intrinsic motivation

A growing body of work in the robotics research community has focused on applying Berlyne's concept of curiosity as an intrinsic motivation for developing skills in robots. Following the encouraging results from the “playground experiment” from Oudeyer et al. (2007) and the advances in self-assessment measures related to novelty and learning progress (Şimşek and Barto, 2004), research has been devoted to the improvement of exploratory behavior and self-development of autonomous agents and robots. Most often these architectures use some evaluation of the progress of the agent in terms of learning, computed as the decrease of the prediction error of the Learning System of the robot (Kaplan and Oudeyer, 2004). Typical architectures modeling curiosity aimed at guiding the exploration of a developing robot often focus on specific task learning problem (Kaplan and Oudeyer, 2005; Luciw et al., 2011) and do not take advantage of the potential availability of humans. However, this principle has also been successfully applied to influence and help a robot in navigation tasks (Hasson and Gaussier, 2010; Jauffret et al., 2013). In this contribution, the authors use self-evaluation measures of success and failure for the robot to express its “frustration” and trigger the help from a human when frustration is too high. They show how this strategy can help the robot subjectively identify deadlock situations, and be assisted in solving a given problem with the help of a human.

#### 1.3.4. Our previous work

Our previous experiments with Aibo robots examined the difference in regulatory behaviors used by the robot, and incidentally their effect on its exploration and learning patterns, when interacting with a responsive human and a non-responsive one. The results showed how a responsive human had a strong influence on the average values of the collative variables collected, to the point that the interventions of the human managed to remove the robot from locations high in novelty and complexity (Hiolle and Cañamero, 2008). Our results also suggested that neither of the extreme strategies of constant responsiveness and no responsiveness were ideal, since at the end of all our runs the robot had learned and classified all the encountered patterns, which kept its arousal always under the lowest threshold, with the effect of making the robot to keep turning fast in the arena in a “bored state,” looking for new features to learn.

Finding an appropriate trade-off between constant responsiveness and no responsiveness that could be suited to the environment in question thus required further investigation. In this paper we investigated how that trade-off could be achieved through the dynamics of mutual *adaptation* between the robot and the caregiver.

In a second experimental setup (Hiolle et al., 2012), we tested the same architecture and embodiment with naive users. The subjects interacted with two robots having two different interaction profiles. These profiles differed, behaviorally, in the amount of human attention and help solicited by the robots as a strategy to regulate the duration of the effect of the comfort in the system. In the “needy” profile, the modulatory effect that the comfort provided by the human had on the level of arousal was short-lived. The results gathered in this second experiment demonstrated that the regulatory behavior produced by this robot requested and elicited human help more often. In the other profile, named “independent,” the modulatory effect that the comfort provided by the human had on the level of arousal lasted longer, leading the robot to explore the environment autonomously for longer following the caregiving responses from the humans during a short-term interaction. The self-rating data from the subjects also showed that on average the subjects preferred interacting with a “needy” profile, deemed more responsive.

Following up on these results, in this paper we endeavor to assess more precisely the influence that these regulatory profiles—“needy” and “non-needy”—have on the exploration of a new environment by the robot, assess how the *responsiveness* of the human can influence the interaction, and combine these two elements to endow the robot with the capability to adapt its affective arousal regulation strategies to the affective responsiveness of the human providing comfort.

## 2. Materials and methods

The model and methods used in this research follow up on our previous work on human–robot attachment dynamics (Cañamero et al., 2006; Hiolle et al., 2012) and non-verbal interaction in human–robot dyads (Hiolle et al., 2010) to investigate the dynamics of arousal and its influence on regulatory and exploratory behavior as postulated by attachment theory and its findings presented in section 1.

### 2.1. Experimental setup

In our previous experiments, an Aibo robot (Hiolle et al., 2012) had to explore and learn the objects present in an environment consisting of toys and objects of different colors, shapes and sizes placed on a children's playmat, an environment inspired by Oudeyer's playground experiment (Oudeyer et al., 2007). The objects were a source of arousal for the robot as a function of their novelty. The level of arousal interacted with the parameters of the learning system, also influencing the observable behavior of the robot, particularly the time it would spend in front of an object while learning it. Depending on the profile given to the robot—either “needy” and overtly expressive or “independent” and not expressive, as set by an internal parameter regulating the threshold that made the robot more or less expressive. A human carer was present in this environment and the robot could “solicit” his/her attention by looking around and emitting barking sounds. The robot would receive “comfort” either when the face of the human appeared on the visual field of the robot, or when its contact sensors were touched.

**Figure 1:**
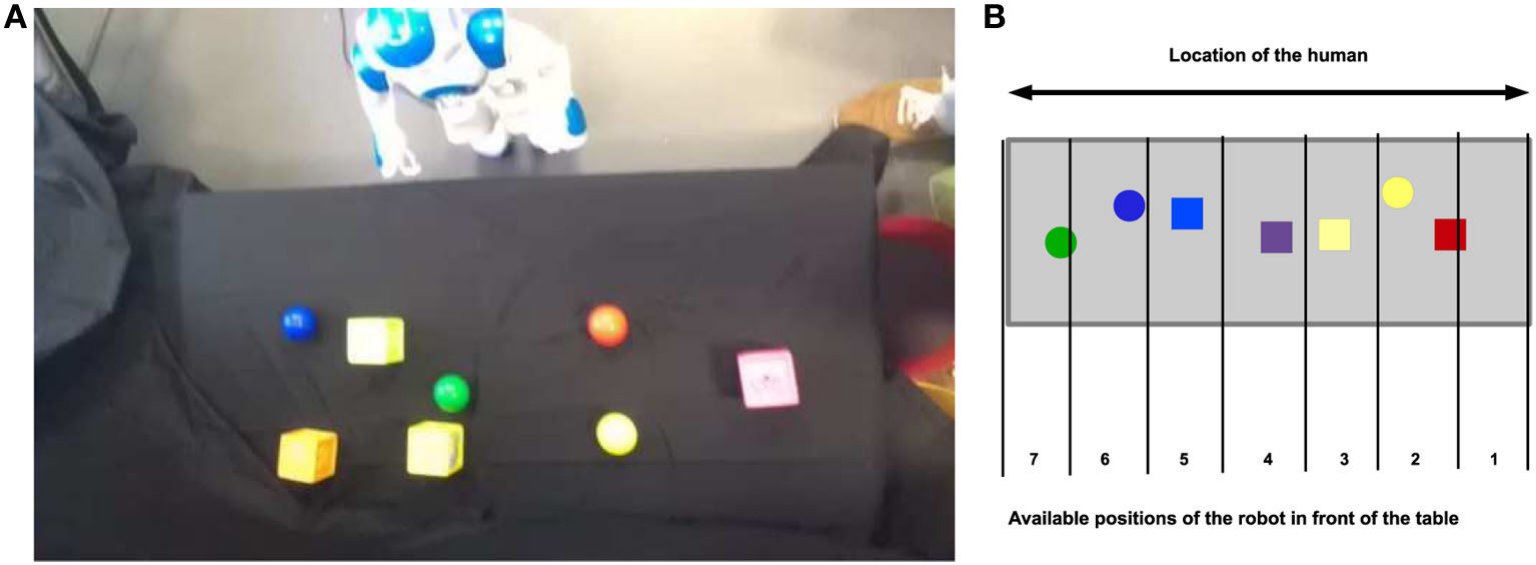
**Experimental setup used with the Nao robot**. Colorful objects are placed on a table covered with a black cloth to facilitate the extraction of the contours of the objects. The robot can then step laterally to change the view of the scene, and the perceptual inputs to be learned. These steps move the robot incrementally from one index position to the next. When the robot reaches end of the table, the direction of the movement is changed, and it then starts moving in the other direction. **(A)** Top view of the table and the robot during the experiment. **(B)** Schematic of the top view of the table and the robot during the experiment with the possible positions and their labels.

In the experimental setup used in the study reported in this paper, the “task” of the robot was similar—exploring and learning novel objects in an environment, for which it can “solicit” (or not) the attention of a human carer as a function of its level of arousal caused by the exploration of the environment, and the carer can provide “comfort” via the visual (by showing his / her face) or tactile (patting the robot on the touch sensor placed on its head) modalities—but we have varied the previous setup as follows. We have used an Aldebaran Nao robot controlled using the architecture described in section 2.2. The robot is placed in front of a table on which several colored objects (toy rubber cubes and balls) are placed as depicted in Figure 1A.

To explore the objects in this environment, the robot moves laterally along the table by stepping first to its left and then to its right. The maximum number of steps in each direction is limited to six, providing the robot with seven different views of the scene as can be seen in Figure 1B. We can therefore record the position of the robot at a given time step and the value of its internal variables. At every time step, we recorded the following internal values used by the architecture: *Stimulation, Arousal, Position, behavior produced*, and *Comfort*.

The robot is connected, via ethernet, to a computer where visual processing and learning are performed, and communicates with the computer using the URBI middleware (Baillie, 2005). The Arousal and Comfort System are running on board using the Urbiscript language. Each iteration of the perception-action loop lasts 300 ms on average, the robot transmits the image from the camera to the computer, where the perception system extracts the contours from the image, transmits them to the Learning System, and then the *Stimulation* value is computed. This value is then sent to the robot to compute the arousal level.

### 2.2. Robot architecture

**Figure 2:**
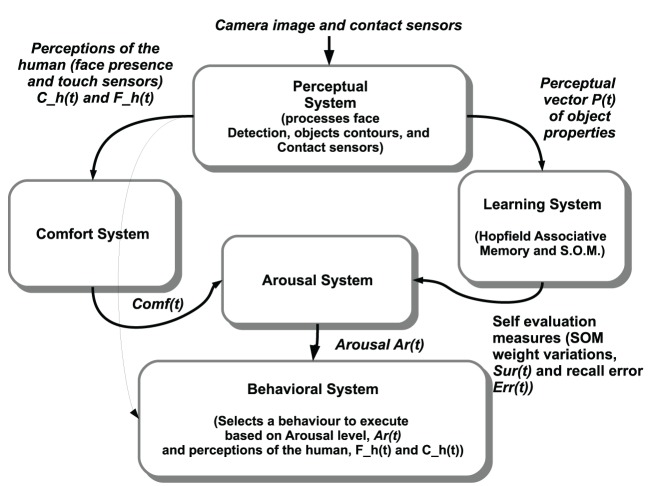
**Components of the robot architecture used in the experiments**. The Perceptual System processes the image from the camera and the contact sensors. In turn, a binary perceptual vector *P*(*t*) containing the properties of the objects is used as input for the Learning System. The Perceptual System also computes the perceptions related to the human: the presence and location of a human face in the visual field, and the perception from the contact sensors of the robot. The self-evaluation measures from Equation (2) from two neural networks in the Learning System regarding the current perception *P*(*t*) are used to compute the level of arousal of the robot. The Comfort System uses the tactile and visual perception of the human (*C*_*h*_(*t*) and *F*_*h*_(*t*), respectively), and the comfort evaluated is used to decrease the arousal level. The Behavioral System uses the *arousal* level and the perceptions related to the human to trigger either requests for assistance when the arousal level is high (i.e., looking for a human and gazing at him/her), walking away in order to explore further when the arousal is low, or remaining still attending to and learning the current perceptual pattern when the arousal is at medium level.

Our architecture is divided into five main components as depicted in Figure 2. The Perceptual System computes the perceptions based on the sensor readings from the camera image and the contact sensors of the robot. A selection of these perceptions (about the human and the other features of the environment) serve as inputs to the Learning System. This allows the robot to try and learn the current features of the environment and permits the evaluation of the novelty of these features. The evaluation measures from the Learning System are fed into the Arousal System, which in the current version of the architecture reflects the dynamics of learning and perceptual novelty. This provides a real time arousal level which, in the absence of any human intervention, correlates with the subjective novelty and complexity of the current situation. Perceptions related to human interventions (distal or proximal), such as the presence of a human face in the visual field or tactile contact on the head sensors, are passed on to the Comfort System. The Comfort System inputs to the Arousal System to decrease the arousal level of the robot in a way akin to the soothing and regulatory effect that the comfort provided by a human caregiver has on an infant (Feldman, 2003). The arousal level is then used by the Behavioral System as input to perform behavior selection and decide whether to explore the environment, remain focused on the current perceptions, or trigger a regulatory behavior to obtain help from the human. The algorithm executed by the architecture is summarized in Algorithm 1.

**Algorithm 1:**
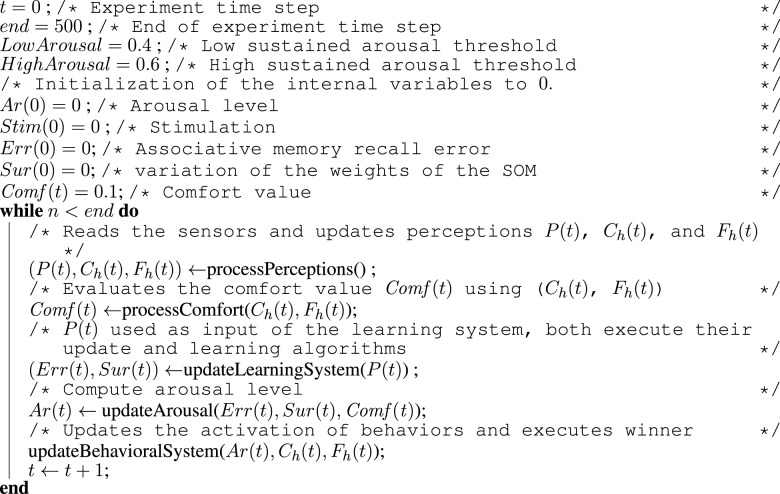
**Algorithm for the entire architecture**.

#### 2.2.1. Perceptual system

The Perceptual System of the robot uses the image from the camera and the contact sensors located on the head of Nao to process information about the objects and humans around it. Perceptions feed into two different components of the architecture—the Comfort System and the Learning System.

**Figure 3:**
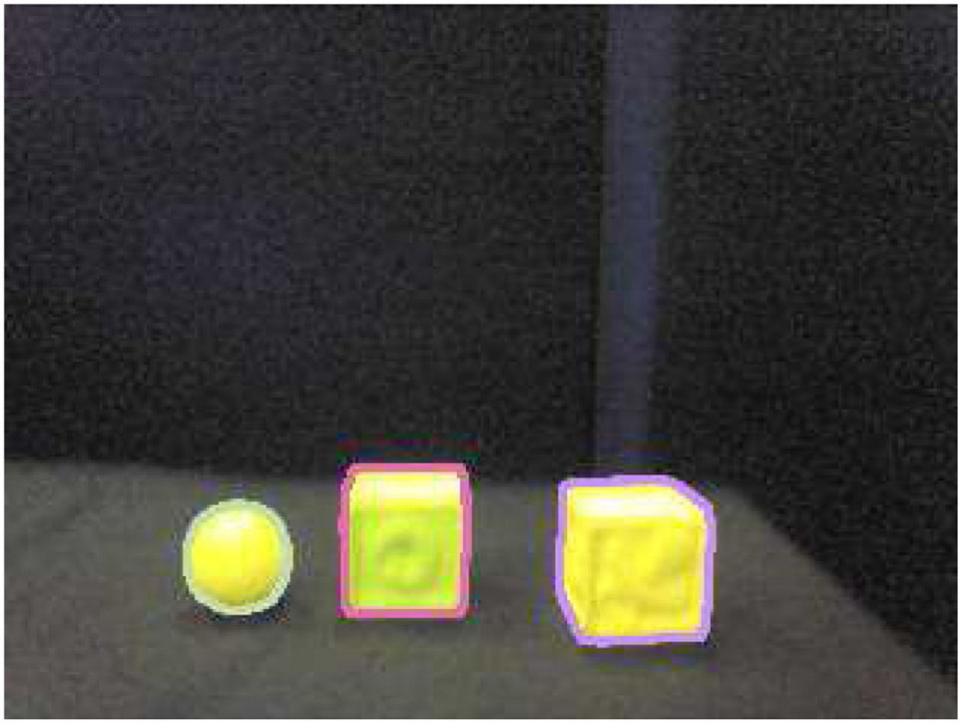
**Contours extracted from the image**. We used a Canny filter as implemented in the OpenCV (version 2.4) library.

Perceptions about objects are extracted from the camera image and provide input to the Learning System. To perform the visual perceptions of the objects, the Perceptual System extracts the contours in the image for the robot to learn features of the visual scene. To this end, we have used available visual processing tools from the OpenCV library. Our algorithm then selects the three largest closed contours using a Canny filter, as depicted in Figure 3, and extracts the following information from them. For each contour, the following properties are calculated to construct a binary vector *P*(*t*):

- The size of the area enclosed in the contour is measured as an integer in the interval [0, 1000].
- The length of the perimeter of the contour is evaluated as an integer in the interval [0, 1000].
- The location (*x, y*) of the centroid of the contour is calculated as a vector of two integers in the interval *x* ∈ [0, 320] and *y* ∈ [0, 240].
- The average of the three color channels in the RGB color space is computed for the enclosed area of the contour, resulting in three floating point values in the range [0, 255].
- The seven values resulting from the previous steps are then normalized and discretized into 50 bins to construct a vector of 350 binary components *P*(*t*) which is used as input to the Learning System.

Perceptions concerning human interventions might come from the camera or the contact sensors and provide input to the Comfort System and the Learning System. To be able to process the input from the human, the Perceptual System contains variables related to the presence of a face in the visual field (*F*_*h*_(*t*)), and the values of the contact sensors (*C*_*h*_(*t*)) located on the head of Nao. The presence of the face is a binary signal updated using the available face detection algorithm from the OpenCV library. The three contact sensors located on the head of the robot are also binary sensors, and are accessed and read using the URBI middleware (Baillie, 2005). Both perceptions take in the values of the sensors in real time and then apply to these values an exponential decay (with a decay rate 0 < α_*sensor*_ < 1) as follows:

(1) $$Perc(t)=\{\begin{array}{ll} sensor(t) & \text{if }sensor(t)=1\\ \alpha_{sensor}\cdot Perc(t-1) & \text{otherwise} \end{array}$$

These choices have been made so that the architecture can keep a trace of the value of each sensor (*sensor(t)*) and a short-term trace of their activity, and to produce continuous (instead of binary) perceptions. The architecture processes these equations to process the perceptions *C*_*h*_(*t*) (activation of the contact sensors on the head of Nao, normally done by a human) and *F*_*h*_(*t*) (presence of a human face in the visual field).

#### 2.2.2. Learning system and self-evaluation measures

As in the architecture used in our previous work (Hiolle and Cañamero, 2008; Hiolle et al., 2012), the robot learns selected features of the scene using two neural networks—a Hopfield associative memory (Davey and Adams, 2004) and a self-organizing map (Kohonen, 1997). These two types of learning algorithms were chosen for the following two reasons: first, their dynamics are well understood, and second, each algorithm provides two different but complementary capabilities associated with the task of learning: classification and recall. The self-organizing map tries to classify the current binary vector, and the Hopfield network converges to the pattern closest to the input vector. At every time step, a new perception vector *P*(*t*) is presented as an input to the two networks, and an iteration of update and learning is performed by both neural networks. The associative memory is updated as described in Algorithm 2, and the self-organizing map is updated using the algorithm presented in Algorithm 3.

**Algorithm 2:**
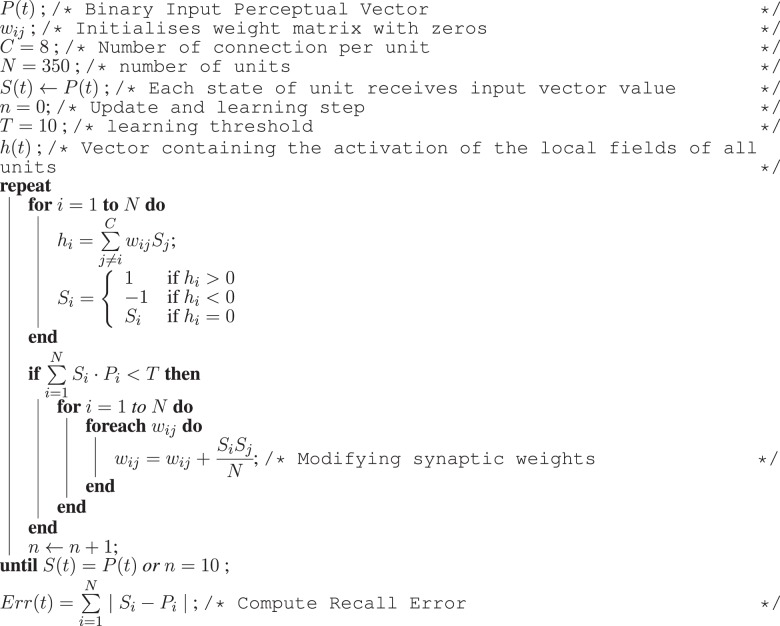
**Algorithm for the update and learning stages of the associative memory**. At every time step *t*, the binary pattern P(t) (once transformed to the [−1, 1] value range required by the associative memory system) is fed to the network in order to be learned. The memory iterates until the all local states *S*(*t*) are equal to *P*(*t*) or after 10 iterations. After this phase, the recall error *Err(t)* is computed.

**Algorithm 3:**
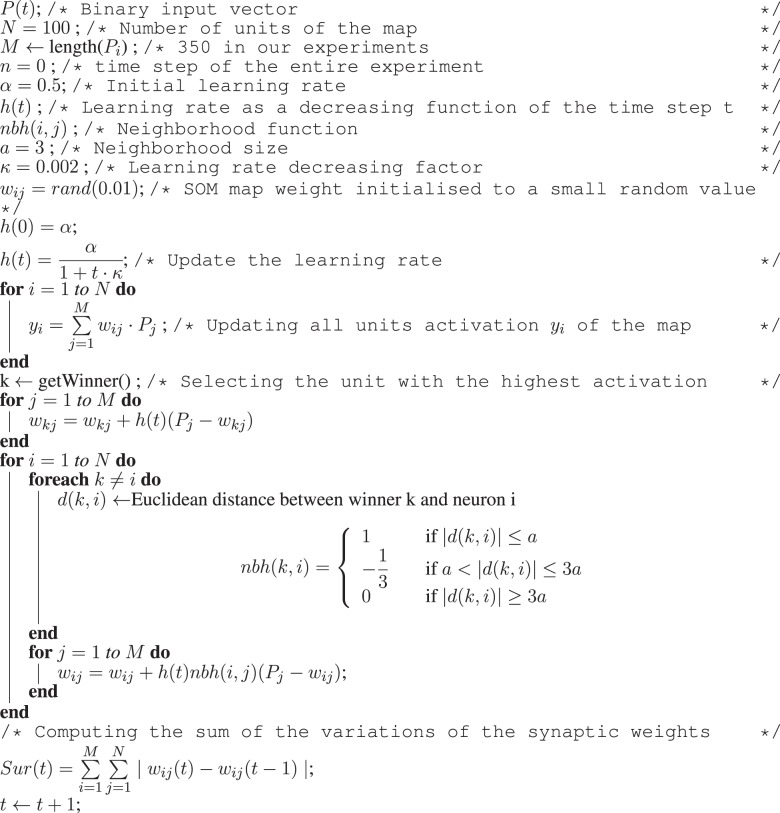
**Algorithm for the update and learning stages of the Kohonen map**. At every time step *t* of the experiment, the binary vector *P*(*t*) is fed as input to the self-organizing map. Then, a learning step is performed as in the original algorithm from Kohonen (1997). After these two steps, the value *Sur(t)* is computed reflecting the variations of all the synaptic weights for this time step.

A real-time measure of the performance of the two networks is produced and stored as an internal variable named *Stimulation, Stim(t)* in Equation (2), which is used to increase the level of arousal. *Stimulation* is computed as the half of the sum of the discrepancy between the pattern recalled by the Hopfield network *Err(t)* (recall error), and the sum of the variation of the weights of the self-organizing map *Sur(t)*.

(2) $$Stim(t)=\frac{Err(t)+Sur(t)}{2}\text{ with }\{\begin{array}{l} Err(t)=\sum_{i=1}^{N}|S_{i}-P_{i}|\\ Sur(t)=\sum_{i=1}^{M}\sum_{j=1}^{N}|w_{ij}(t)-w_{ij}(t-1)| \end{array}$$

In Equation (2), the intermediate variable *Err(t)* is the discrepancy between all *N* components of the recalled pattern from the Hopfield network (*S*_*i*_) and the current pattern (*P*_*i*_), *N* is the number of components of the input vector, and *M* the number of units in the self-organizing map. The value of *Err(t)* indicates how novel the current perception *P*(*t*) is for the memory, since the more novel it is, the higher the recall error *Err(t)* will be. The second term, *Sur(t)*, is the sum of the variations of the synaptic weights of the Kohonen map. This value reflects how far the current synaptic weights of the winning unit are from the current perception. This measure also reflects the novelty of the current perceptions *P*(*t*). These two measures are related to the prediction error used in other systems for self-evaluation (Cohn et al., 1994; Weng, 2002).

#### 2.2.3. The arousal system and the comfort system

The arousal model is an adaptation of the model described and studied in previous work (Hiolle et al., 2012). The arousal level increases as a function of the *Stimulation* perceived, to reflect the cognitive effort demanded by the current situation and the familiarity of the current perceptual vector *P*(*t*). The arousal is modeled as a smooth average of the *Stimulation*, which is a real-time evaluation of the recall error of the associative memory and the variation of the synaptic weights of the self-organizing map. Additionally, in the same way as arousal and distress are modulated by the attachment figure in infants, the robot's caregiver can decrease the arousal via tactile contact or by presenting his/her face in the visual field.

(3) $$Ar(t)=\{\begin{array}{ll} \frac{\tau_{ar}\cdot Ar(t-1)+Stim(t)}{\tau_{ar}+1} & \text{if }Comf(t)\leq 0.1\\ Ar(t-1)-\alpha_{ar}\cdot Comf(t) & \text{otherwise} \end{array}$$

As we can see in Equation (3), the arousal level is a scalar value computed as an exponential average of the stimulation perceived when no comfort *Comf(t)* is perceived. Exponential averaging is used to prevent sudden changes that could lead to abrupt changes in the behavior of the robot. The window parameter τ_*ar*_ controls the influence that the current *Stimulation* has on the arousal, thus defining its slope; it is a smoothing factor that biases this influence either toward “the past” (a larger τ_*ar*_ that produces smoother behavior) or toward “the present” (a smaller τ_*ar*_ that gives rise to more reactive behavior), as a function of the variability of the *Stimulation*.

*Comf(t)* is the internal variable evaluating the influence of the human, i.e., the comfort provided through the two available modalities, which are the perception of the head contact sensors (*C*_*h*_(*t*)) and the perception from the face detection module (*F*_*h*_(*t*)). *Comf(t)* is calculated as follows:

(4) $$Comf(t)=\{\begin{array}{ll} \frac{Comf(t-1)\cdot \tau_{h}+C_{h}(t)+F_{h}(t)}{\tau_{h}+1} & \text{if }C_{h}(t)>0\\ & \text{or }F_{h}(t)>0\\ \beta_{h}\cdot Comf(t-1) & \text{otherwise} \end{array}$$

The trace rate β_*h*_ controls the rate at which the past comfort subsists in the system and provides the architecture with a means to vary the duration of the effect of human interventions. τ_*h*_ controls the weight given to past perceived comfort as it defines the time window on which the comfort value is updated. Both parameters were used in our previous HRI study (Hiolle et al., 2012) to define the two robot profiles that react differently to human interventions. A “needy” robot, with low β_*h*_ and τ_*h*_ values, has a short-lived *Comf(t)* that results in more frequent increase of arousal in the face of novelty, and therefore calls for human comfort more often. An “independent” profile, for which the *Comf(t)* decays more slowly and therefore decreases the arousal level for a longer period of time, calls for human comfort less often.

#### 2.2.4. Interactions between the arousal system, the comfort system, and the learning system

The interaction between the arousal-comfort interplay and learning are as follows. Following the models discussed in section 1.2.4, a moderate level of arousal fosters learning, while extreme (high and low) levels of arousal hinder learning. An excessively high level of arousal reflects the lack of “stability” in the underlying neural networks, leading the robot to stop in front of the stimulus currently perceived (the source of arousal) and “call for help” (look for a human). An excessively low level of arousal reflects “boredom” (lack of stimulation, lack of novel input to the networks) that leads the robot to divert attention away from the current stimulus and explore in search of new ones. The fact that the level of arousal descends from high to medium (when the decrease is not directly produced by human comfort) indicates that the robot is learning new, “interesting” things, and in fact a medium level of arousal following a high level is a sign that the robot has learned something new. The fact that the level of arousal descends from medium to low indicates that the robot is perceiving stimuli that are already familiar and have low “interest.”

The interactions between arousal levels, the comfort provided and the way this is differentially processed by the “needy” and “independent” robots, and the learning system, leads to different exploration and learning dynamics in the two robots, as we incidentally observed in our previous work (Hiolle and Cañamero, 2008). In general terms, we previously observed that, in the face of high novelty, a “independent” robot would explore more than a “needy” robot, and a “needy” robot would spend more time attending to the current stimulus and learning its features than an “independent” one. The first two experiments presented in this paper were designed to investigate whether the same phenomenon would be observed using a different robot embodiment and a different environment, and to better understand and assess its mechanics and dynamics.

#### 2.2.5. Action selection and the behavioral system

**Figure 4:**
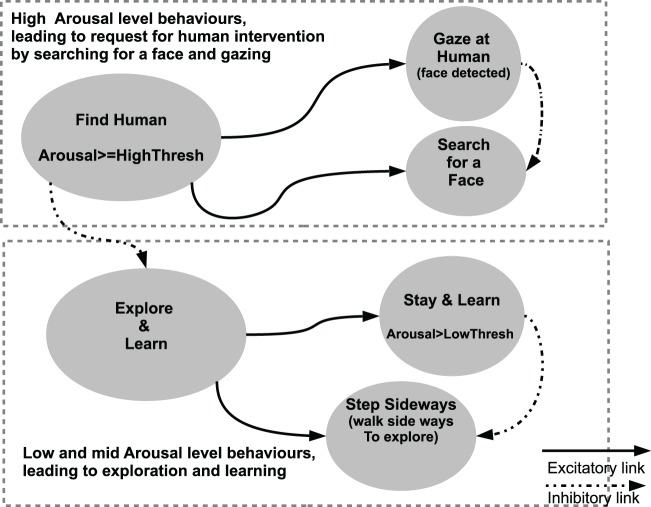
**Behaviors used and their connectivity**. The two behavioral systems “Explore-and-learn” (exploratory behavior) or the “Find-Human” (the main regulatory behavior) are mutually inhibiting. If the Arousal level is above than *Highthresh*, the regulatory behavior is activated and in turn inhibits the exploratory behavior. The “Explore-and-learn” behavior, when active, activates the two connected behaviors “Stay-and-Learn” and “Step-Sideways.” The activation of the behavior “Stay-and-Learn” is modulated by the Arousal level. If the Arousal level is above *LowThresh*, the behavior “Stay-and-Learn” maintains a high activation level and inhibits the “Step-Sideways” behavior. If the Arousal level is lower, the activation of the behavior “Stay-and-Learn” is null, and the behavior “Step-Sideways” is not inhibited and therefore executed. In a similar process, the regulatory behavior “Find-Human” either search for a face when the face detection algorithm does not detect one, or tracks a face and gaze at the human. The perception of a face in the visual field modulates the behavior “Gaze-at-human,” which inhibits the behavior “Search-Face.”

The Behavioral System, depicted in Figure 4, takes inspiration from behavior-based-robotics approaches, particularly (Brooks, 1986; Cañamero, 1997; Arkin, 1998; Avila-Garcia and Cañamero, 2004) and contains a set of predefined behaviors to be executed depending on the current perceptions and the arousal level.

Each behavior possesses its own activation level, which reflects the relevance of that behavior for the current situation and is computed based on the Arousal level and behavior-related perceptual information. In a similar vein to Avila-Garcia and Cañamero (2004), our simple architecture implements in effect a two-resource action selection problem. Our robot must choose between two activities, “Explore-and-Learn” and “Find-a-Human” using a Winner-take-all action selection algorithm (Avila-García et al., 2003). The activation of these behaviors only depends on the level of arousal. If the arousal is greater than or equal to a given threshold (which here we have chosen to set to a high level, *Highthresh*), the behavior “Find-a-Human” will be executed. These two main behaviors can trigger other simpler behaviors, also following a Winner-take-all policy. The “Explore-and-Learn” behavior selects whether to attend to and learn the current stimuli (“Learn” behavior), or to move away from it and explore other elements of the environment (“Explore” behavior). The regulatory behavior “Find-Human” can either trigger the appetitive behavior to search for a face by moving its head (and therefore the camera located on its head), or the consummatory behavior of tracking a face (using the location of the face in the visual field provided by the perceptual system).

### 2.3. Experiment design

Building on two strands of previous work that assessed independently (a) the differences between two “idealized” robot profiles—a “needy” and an “independent” robot—in terms of their use of a caregiver as a means to regulate the “stress” (arousal) produced by the exploration and learning of a novel environment (Hiolle et al., 2012), and (b) the effects on the robot behaviors of two caregiver profiles varying in their responsiveness—“responsive” and “non-responsive”—to the regulatory requests of the robot (Hiolle and Cañamero, 2008), in this paper we bring both strands together and take a step further by having the robot adapt its regulatory behavior along the “needy” and “independent” axis as a function of the varying responsiveness of the caregiver. A different robot platform—a Nao robot, rather than the Aibo robot used in previous work—was also used this time.

#### 2.3.1. Two robot profiles

As in our previous work (Hiolle et al., 2012), we have used two different robot behavioral profiles, varying in the way they regulate high levels of arousal: a “needy” and an “independent” robot, borrowing the terminology commonly used in attachment theory regarding regulatory behavior.

From a behavioral perspective, the “needy” robot “solicits” human attention often, whereas the “independent” robot seldom does it.

From an architecture viewpoint, these profiles process the interventions of the human, and therefore the comfort provided, with different temporal dynamics. In terms of the architecture, the profiles vary in terms of the temporal parameters used to compute the variable *Comf*, namely the length of the time window τ_*h*_, and the trace rate β_*h*_. The “needy” profile uses a short time window τ_*h*_ and a low trace rate β_*h*_ as in Equation (4). Having low values for these two parameters leads to a shorter-lived *Comf*. This in turn means that the robot will call for assistance often and therefore fits with the “needy” characteristics in terms of attachment behavior. Higher values for these parameters, implemented in the “independent” profile, produce fewer calls for attention and a longer effect of the comfort on the level of arousal. From an observer's point of view, naive humans interacting with this robot also tend to qualify it as being more independent (Hiolle et al., 2012). The parameters used for the two profiles are presented in Table 1.

**Table 1:** **Parameters used in the experiment for the “needy” and the “independent” robot profiles**.

**Parameter name in the model**	**“Needy” profile value**	**“Independent” profile value**	**Description**
α_*ar*_	0.6	0.6	Decay rate of the arousal
τ_*ar*_	5	5	Time window for the level of Arousal
τ_*h*_	3	8	Time window for the level comfort
β_*h*_	0.7	0.95	Trace rate of the comfort
*HighThresh*	0.6	0.6	Higher threshold for the level of Arousal
*LowThresh*	0.4	0.4	Lower threshold for the level of Arousal

#### 2.3.2. Caregiver responses

In order to compare the two profiles and the dynamics they produce in a highly controlled and systematic way, we designed an automated system to produce the responses of the caregiver. A “caregiving” response is produced every time the behavior “Find-Human” is activated, precisely one second after the behavior is activated, which is a good approximation (empirically established) to the time a human present by the setup takes to respond to the robot. The mechanism to produce this “caregiving” response consists of modifying the variables that monitor the presence of a human face (*F*_*h*_(*t*)) and contact on the touch sensor on the head (*C*_*h*_(*t*)), and hence to produce *Comf*.

Although this system can generate any caregiving profile between the two extremes of constant responsiveness and non-responsiveness, or between constant presence and total absence, only an immediately responsive caregiver (which responds to each request from the robot) was used for these tests, since this is the profile that modulates the arousal to a greater extent. Let us note, however, that immediate responsiveness can give rise to two different caregiving styles when interacting with different robot profiles, and that match them: a constantly present caregiver when interacting with the needy robot, and a more “relaxed” or “hands-off” caregiver when interacting with the independent.

In contrast with our previous studies (Hiolle and Cañamero, 2008), where the responsive caregiver was a real human (the experimenter), and therefore the timing of the responses was subject to some amount of noise depending on the location of the robot, this automated response system provides us with clear caregiving patterns in terms of the timing and frequency of the responses.

## 3. Experiments and results

With the aim of studying how a robot that explores and learns novel environments can adapt its affective regulatory behavior to that of a human “caregiver” as a function of the varying responsiveness of the caregiver, the purpose of our experiments was to investigate the following research questions:

(Q1) Whether and how different caregiving styles can be suited to different characteristics of the “infant” (robot) in terms of strategies used to regulate stress, i.e., to the characteristics of the two different robot profiles envisaged (experiment 1).
(Q2) Whether and how different types of interaction and “caregiving profiles” might affect differentially the cognitive and affective development of the “infant” (robot)—namely its regulatory, exploratory and learning patterns (experiment 2).
(Q3) The use of adaptation as a mechanism to produce a suitable match between different robot profiles and caregiving styles (experiment 3).

### 3.1. Experiments in a simpler environment

**Figure 5:**
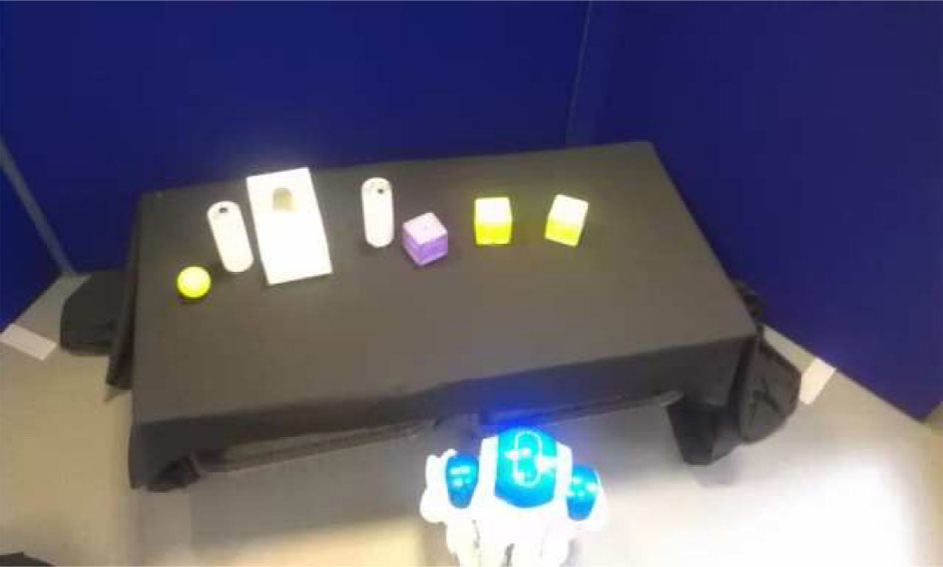
**The simpler environment used in the initial tests of the robot profiles**.

We first tested the two robot profiles in a simpler environment, with only a few objects placed on the table as can be seen in Figure 5.

We tested each profile in 10 runs (thus giving a total of 20 runs in this environment) using the automated “responsive” caregiver profile that responded to each request from the robot. For each profile, results and overall duration were very similar across all runs. We provide here results from a representative example.

**Figure 6:**
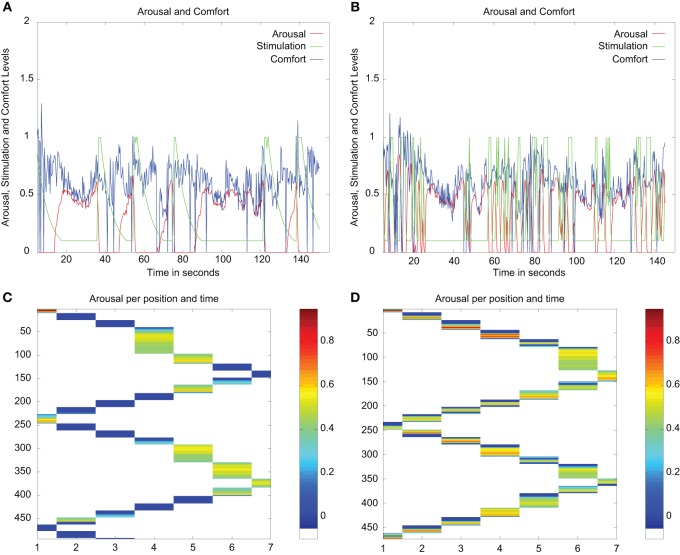
**Summary of the main variables of the architecture from experimental runs in the simple environment for both profiles of the robot**. Panels **(A,B)** display show the arousal variations and the variables used to compute the arousal level. Panels **(C,D)** show the arousal of the robot against time for each position that the robot can reach in the environment. **(A)** Evolution of the Arousal (red), Comfort (green), and Stimulation (blue) for the “independent” robot. **(B)** Evolution of the Arousal (red), Comfort (green), and Stimulation (blue) for the “needy” robot. **(C)** Temperature plot representing the level of arousal as a function of time (*y* axis measured in timesteps) and position (*x* axis) for the “independent” robot. **(D)** Temperature plot representing the level of arousal in function of time (*y* axis measured in timesteps) and position (*x* axis) for the “needy” robot.

The results for each robot profile in a run of 150 s are displayed in Figure 6. As we can see in Figure 6A,B, the main difference between the profiles is the amount of comfort that each robot requested. In these figures, this difference is illustrated by the number of peaks in the *Comf* (in green) which are much more numerous for the “needy” profile, as predicted by the model. We can also see on the graphs for the “independent” robot the difference in the lasting effect of the value of *Comf*, showing a trace lasting up to 10 s. This effect reduces the arousal to a low level, and this drives the robot to move and explore. In terms of exploration, the lasting effect of the comfort provided increases exploration time since the robot stops to attend to the stimuli after longer periods of exploration, i.e., it stops less often than under high arousal. During exploration periods, the stimuli perceived (the contours of the available objects) vary faster in their location in the visual field, their subjective size (area and length of contour perceived), and most likely their color in the RGB space as the angle of view differs.

The “Temperature” plots in Figure 6C,D represent the arousal level against time and position in the setup. They show how long each robot profile spent at each position in the setup. In these figures, we can see that, on average, both profiles go through the setup at a similar pace. Starting from the first position (labeled 1), they reach the end of the table around time step 160 (approximately 50 s), and come back to the other end of the table by time step number 250 (approximately 83 s). This shows how (the parameters of the model processing the *comfort* level in) *each of the two profiles require a different caregiving style in order to obtain a comparable exploration dynamics*.

In the figures, we can also observe that the “needy” profile shows more frequent episodes of medium arousal (for instance in time step 50–100, and again at time step 300) than the “independent.” The “independent” profile shows these episodes less often since the longer lasting effect of the comfort provided reduces its arousal to a low level for longer. Our second experiment aimed to investigate the potential implications of this difference.

In conclusion, while both robot profiles took approximately the same time to walk through the environment, their regulatory behaviors and patterns of exploration were different. While the “needy” robot needed frequent comfort from the caregiver to be able to learn and progress, the “independent” robot used less comfort to learn and progress the same amount. In other words, answering our research question Q1, to achieve the same results with needy and independent profiles, different caregiving styles are needed.

### 3.2. Experiments in a more complex environment

**Figure 7:**
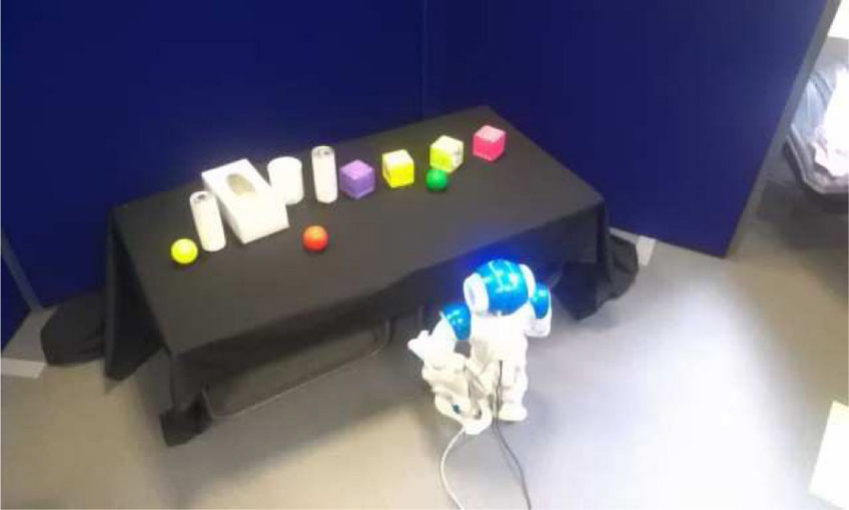
**The more complex environment used in the second set of tests of the robot profiles**. More objects that vary in shapes and sizes were placed on the table.

Our second set of experiments aimed at testing how the same robot profiles, with access to the same caregiving style as previously, would cope with their arousal levels and explore and learn the environment under more challenging conditions. To achieve this, we put the robots in a similar but more crowded setup (see Figure 7), where the higher number of objects increased the complexity of the exploration and learning task. This modification of the setup would allow us to assess, for each robot profile and with equally responsive caregivers as previously, how the dynamics of the exploration/learning and generally the behavior of the robot is influenced by the increased density of new percepts in the environment, and hence by the arousal and its differential processing in each profile.

We tested each profile in 10 runs (thus giving a total of 20 runs in this environment) using the automated “responsive” caregiver profile that attended to each request from the robot. For each profile, results were very similar for all runs as was the overall duration. We provide here results from a representative run that lasted approximately 160 s.

#### 3.2.1. Comparison to the simpler environment

**Figure 8:**
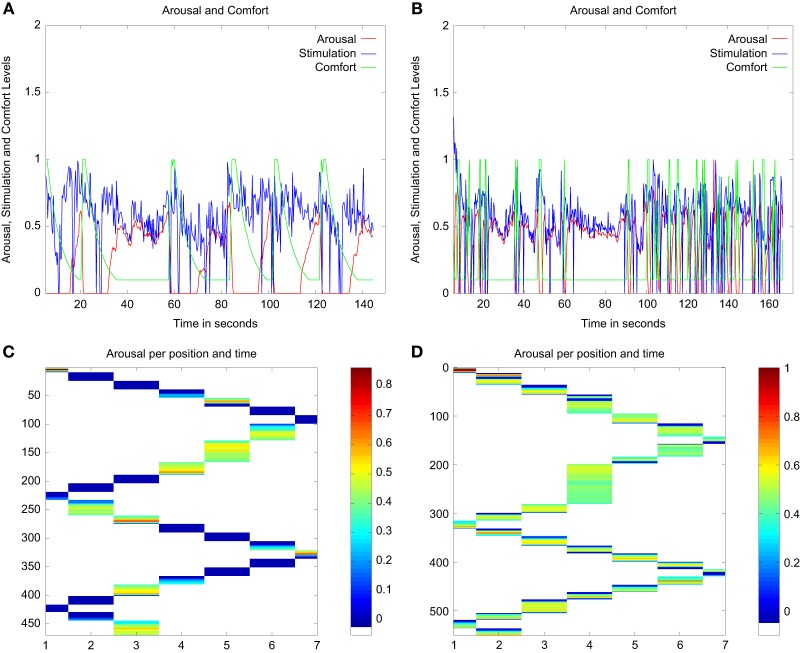
**Summary of the main variables of the architecture from experimental runs in the more crowded environment for both robot profiles**. Panels **(A,B)** show the variations in arousal and the variables used to compute the arousal level. Panels **(C,D)** show, for each robot profile, the arousal of the robot at each time for each position of the setup the robot can reach. **(A)** Evolution of the Arousal (red), Comfort (green), and Stimulation (blue) for the “independent” robot. **(B)** Evolution of the Arousal (red), Comfort (green), and Stimulation (blue) for the “needy” robot. **(C)** Temperature plot representing the level of arousal as a function of time (*y* axis measured in timesteps) and position (*x* axis) for the “independent” robot. **(D)** Temperature plot representing the level of arousal as a function of time (*y* axis measured in timesteps) and position (*x* axis) for the “needy” robot.

In comparison to the simpler environment, the increased complexity of this environment had a higher impact on the “needy” than on the “independent” robot, both in terms of the trade-off between learning and exploration and in terms of the regulatory behaviors produced. We can observe in Figure 8 that the “needy” robot explored the setup at a considerably slower pace than it did in the simpler environment over the whole run, since it was confronted with more situations where the stimulation (and hence the arousal) increased due to the novelty of the perceived objects and their properties. The “independent” robot also showed more periods of high arousal (time step 170 and 270) and longer periods of medium arousal than it did in the simpler setup (cf. Figures 6C, 8C). In terms of *Comf* needed, the “independent” profile did not solicit the attention of the caregiver more often than in the simpler environment. Once more, the longer-lasting effect of the comfort appears to have been sufficient for the robot to go through this setup in a similar manner as through the simpler one. For the “independent” profile, the simpler and more complex setup were equivalent in terms of exploratory behavior, since the longer-lasting effect of the comfort provided made the robot move and explore. On the other hand, the “needy” profile produced more regulatory behaviors than in the previous scenario, as expected by the increased density of available objects. Despite receiving the more frequently requested comfort, due to the effects of more demanding setup, the “needy” robot also showed longer periods of medium arousal than before since even the increased comfort was not sufficient to decrease the arousal below medium levels, i.e., to the low threshold that fosters exploration.

#### 3.2.2. Comparison between the two profiles

A comparison of both profiles in this more complex environment given similar responsiveness from the caregiver (responsiveness to every request from the robot for both profiles, following the same responsiveness pattern as in the simpler environment) shows additional differences than those found in the simpler environment. Differences between the two profiles were found in terms of exploratory behavior and learning dynamics. Due to the higher density of objects and features, again in this environment the “needy” robot requested assistance more often than the “independent” and therefore remained in the same position for longer (even after comfort was provided) due to the interplay between the stimulation perceived and the comfort provided. However, contrary to what happened in the simpler environment, both profiles explored this more complex environment at different paces. The “needy” robot explored this time at a slower pace than the “independent”: while the “independent” robot took 140 s to walk through the setup twice, the “needy” took 170 s to do the same.

Contrary to what might seem on a first approximation, these results do *not* suggest an advantage of the “independent” profile over the “needy”; they merely indicate a *difference* in the way both robots explore and learn. Due to the differential interactions between arousal levels, the comfort provided, and the learning system (cf. section 2.2.4), the fact that the “needy” robot spent prolonged periods in front of a novel stimulus, and that its arousal descended from high to medium levels while doing so, means that it spent more time learning and that it learned “more carefully” the features of the novel objects (the learned patterns were better consolidated in the underlying neural networks). Comfort provided by the human as requested by the robot when the level of arousal was very high reduced the level of arousal by a small amount that helped the robot to focus its attention on the novel stimulus, bootstrapping the learning process. The longer-lasting effects of human-provided comfort on the “independent” robot generally kept its level of arousal in the medium-low range, fostering exploratory behavior to the detriment of time spent learning objects.

In other words, confirming incidental observations that our previous work had suggested, in this experiment we found that while the “independent” robot explored more, the “needy” robot learned more. Neither robot profile is at an absolute advantage with respect to the other. From the point of view of the robot (e.g., in terms of performance or task-execution) both, a more exploration-oriented and a more learning-oriented behavior, can present advantages and disadvantages depending on the specific circumstances or the task to which the robot is confronted. From the point of view of human–robot interaction, both profiles are also equally valid and potentially useful, since each might better suited to different types of human profiles and preferences. Answering our research question Q2, our results show that different types of interaction and “caregiving styles” affect differentially the regulatory, exploratory and learning patterns of the two robot profiles. The interaction dynamics between the immediate responsiveness of the caregiver and each profile gave rise to a responsive and constantly present caregiver in the case of the “needy” robot, and to a responsive but more “hands off” caregiver in the case of the “independent” robot. The profiles of the robots and the caregiving styles matched to give rise to different but equally valid regulatory, exploratory and learning patterns.

Ideally, a robot should be able to behave according to both profiles in an adaptive way that is appropriate for the task, the environment, or the human user concerned. Our third set of experiments was designed to test a mechanism to permit switching between profiles to adapt to a human as a function of his/her interaction preferences, and assess the implications of this adaptation for the robot and the dyadic human–robot interaction.

### 3.3. Experiments with varying responsiveness of the caregiver: affective adaptation

As the results previously presented show, the exploration and learning of the robot are influenced both by the behavior of the caregiver, and by the parameters used to compute the comfort level and its influence on the arousal level. The experiments conducted in our previous work tested “idealized” categories of caregiving styles showing clearly defined profiles at opposite ends of the “responsiveness” dimension—a constantly present caregiver and a mostly absent caregiver. However, in real-world interactions with humans, those clear “typical” profiles tested are unlikely to be found: people are more likely to show a profile somewhere between those extremes, and the same person might also change his/her *responsiveness* over time. At the same time, people might vary in their preference for a more “needy” or a more “independent” robot at different points in time. A robot interacting in the real world should thus be able to adapt its behavior to the changing interaction styles (in the case concerned in this paper, in terms of responsiveness) and preferences of the human. The adaptation that we are concerned with here is a case of *affective adaptation* that relies on the assessment of the behavior of the caregiver when help and attention are requested.

Our next step was thus to endow the robot architecture with adaptation capabilities—in this case, permitting the robot to assess the *responsiveness* of the human to the robot's regulatory behaviors (requests for attention and help), and vary those regulatory behaviors (in terms of “independence” or “neediness”) as a function of the responsiveness of the human.

This new element was inspired by the literature on parental caring style and the dimensions used to assess it (De Wolf and van Ijzendoorn, 1997). The notion of *responsiveness* has been linked to a carer's ability to attend to an infant's demands in a timely and accurate manner. The model of the formation of patterns of attachment (Ainsworth et al., 1978) postulate that infants adapt to the interactive style of their caregiver and their “trust” in the caregiver's ability to soothe them influences their behavior.

In essence, in this new condition the architecture correlates the activity of the regulatory behavior of the robot (finding a human by looking for a face) with the comfort received. This correlation is reflected in a new variable *responsiveness, Resp*_*h*_(*t*) (with 0 < *Resp*_*h*_(*t*) < 1), which increases when the robot receives comfort after having made a request. The *responsiveness* is computed as shown in Equation (5) when the behavior “Find-Human” is active:

(5) $$Resp_{h}(t)=\{\begin{array}{l} Resp_{h}(t-1)+\alpha_{resp}\cdot (1-Resp_{h}(t-1))\\ \text{ if }Comf(t)>0.1\\ Resp_{h}(t-1)-\alpha_{resp}\cdot (1-Resp_{h}(t-1))\\ \text{ otherwise} \end{array}$$

The two parameters controlling the dynamics of the comfort *Comf(t)*, β_*h*_ and τ_*h*_ (cf. Equation 4), are respectively updated as follows:

(6) $$\beta_{h}=0.7+(1-Resp_{h})\cdot R_{\beta_{h}}$$

and

(7) $$\tau_{h}=3+(1-Resp_{h})\cdot R_{\tau_{h}}$$

As we can see in Equations (6, 7), the decrease of these two parameters is proportional to the calculated *responsiveness*. The two constants *R*_τ_*h*__ and *R*_β_*h*__ determine the range of variability of the two parameters.

We tested this adaptive architecture in the same setup used in the second experiment (section 3.2) for a total of five runs. However, this time a real human (the experimenter) played the role of the caregiver. To assess the dynamics of the adaptation and its effect on the regulatory and exploratory behaviors of the robot the robot, the experimenter alternated periods of extreme *responsiveness* and periods of low *responsiveness*. The parameters used are presented in Table 2.

**Table 2:** **Parameters used in the adaptive regulation experiment**.

**Parameter name in the model**	**Value**	**Description**
α_*ar*_	0.8	Decay rate of the arousal
τ_*ar*_	3	Time window for the level of arousal
τ_*h*_	3	Initial time window for the level comfort
β_*h*_	0.7	Initial trace rate of the comfort
α_*resp*_	0.03	Variation constant for the responsiveness
*Resp(0)*	0.5	Initial responsiveness level
*R*_τ_*h*__	7	Range of variation of the comfort time window
*R*_β_*h*__	0.3	Range of variation of the comfort trace rate
*HighThresh*	0.6	Higher threshold for the level of Arousal
*LowThresh*	0.4	Lower threshold for the level of Arousal

**Figure 9:**
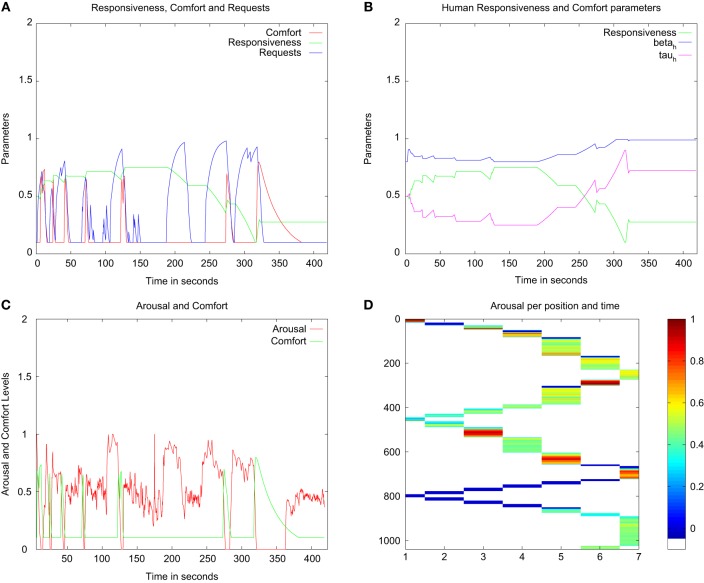
**Effects of the adaptive controller based on the *responsiveness* of the human caregiver**. **(A)** Variations of the *comfort* sensed (in red), the evaluated *responsiveness* (in green) of the human caregiver, and the requests of the robot (in blue). **(B)** Variations of the parameters of the *comfort* evaluation (the trace rate β_*h*_ and the time window τ_*h*_ which is normalized at an eighth ratio for presentation ease), and the evaluated *responsiveness* of the human. **(C)** Variations of the *comfort* (in green) and the *arousal* level of the robot (in red). **(D)** Temperature plot of the Arousal level of the robot as a function of time and the position of the robot.

The results from a typical run of the experiments testing the adaptive architecture are presented in Figure 9. The top left of Figure 9A shows how the evaluated *responsiveness* varied in time depending on the responses of the human caregiver. As we can see from the start of the run, when a request was made, the evaluated *responsiveness* started decreasing since the caregiver had not yet responded. At every peak of the *comfort* level, as projected from the model, the evaluated *responsiveness* steadily increased (approximately from second 5–140). In turn, the parameters used to evaluate the *comfort* level, and therefore to lower the level of *arousal* were updated and decreased. The profile of the robot slowly developed toward a more “needy” one, since the human caregiver responded at every call of the robot. This led the robot toward a more “learning-oriented” behavior over that period. After 150 s, the experimenter stopped responding to the demands of the robot. We can see that during the next two displays of regulatory behavior (at approximately 170, 240 s), the *responsiveness* decreased as a result of the failed attempt to obtain attention. Consequently, as modeled, the *Comfort* parameters varied toward the more “independent” profile. At the end of the run, both in Figure 9C,D, we can clearly see the difference in the lasting effect of the comfort provided by the experimenter (at approximately 320 s). The level of arousal decreased to a low level, driving the robot to explore more.

## 4. Discussion and conclusion

We have presented a robot architecture and experiments assessing the interplay between affective variables—namely the level of arousal of the robot as a function of the novelty and complexity of the environment and the comfort provided by a caregiver to help regulate that arousal—in dyadic robot-(human) caregiver interactions and their effects on the exploratory, learning and regulatory behaviors of the robot.

Building on two strands of previous work that assessed independently (a) the differences between two “idealized” robot profiles—a “needy” and an “independent” robot—in terms of their use of a caregiver as a means to regulate the “stress” (arousal) produced by the exploration and learning of a novel environment (Hiolle et al., 2012), and (b) the effects on the robot behaviors of two caregiver profiles varying in their responsiveness—“responsive” and “non-responsive”—to the regulatory requests of the robot (Hiolle and Cañamero, 2008), in this paper we bring together both strands and take a step further by having the robot adapt its regulatory behavior along the “needy” and “independent” axis as a function of the varying responsiveness of the caregiver. A different robot platform—a Nao robot, rather than the Aibo robot used in previous work—was also used this time.

The robot architecture and the dynamics of the model take inspiration from psychological and neurobiological theories and findings regarding the development of attachment bonds on mother–infant interactions in the early years of life (Sroufe and Waters, 1977; Sroufe, 1995). The architecture models the increase of the level of arousal as an evaluation of the novelty of the stimuli perceived in the environment. The decrease of the level of arousal is a product of the reduction of the novelty (or level of stimulation) perceived and the *Comfort* provided by a human. As postulated and formalized by theories on curiosity and exploratory behaviors in humans and animals (Berlyne, 1954), and optimal arousal control (Anderson, 1990), the level of arousal then drives the behavior of the robot, exploring and seeking novelty when the arousal is at a low level, and displaying a regulatory behavior when the arousal is high. The robot used two neural networks as a Learning System, the stability and accuracy of which were used as a self-evaluation measure of perceived novelty and external stimulation.

Using the two above-mentioned profiles—“needy” and “independent”—the architecture was tested in three experiments investigating the following research questions:

(Q1) Whether and how different caregiving styles can be suited to different characteristics of the “infant” (robot) in terms of strategies used to regulate stress, i.e., to the characteristics of the two different robot profiles envisaged (experiment 1).
(Q2) Whether and how different types of interaction and “caregiving profiles” might affect differentially the cognitive and affective development of the “infant” (robot)—namely its regulatory, exploratory and learning patterns (experiment 2).
(Q3) The use of adaptation as a mechanism to produce a suitable match between different robot profiles and caregiving styles (experiment 3).

The experiments used three variants of a simple setup in which an Aldebaran Nao robot had to learn the features of several objects located on a table.

The first set of experiments in which each of the robot profiles had to explore and learn a simpler environment with a few objects on the table was carried out to examine potential differences between the profiles in terms of exploratory and regulatory behaviors. The “independent” profile needed less interaction with the human caregiver to progress in its exploration. Every time that the caregiver provided comfort to the robot, its longer-lasting effect in the architecture led the robot to progress faster and farther in the setup. In contrast, for the “needy” profile to progress with comparable dynamics, the caregiver needed to have almost constant presence to respond to the demands of the robot. The results thus showed that, to achieve the same results with the two robot profiles, different caregiving styles are needed.

A second set of experiments increased the perceptual complexity of the environment, affecting the dynamics of arousal increase and regulation. The two robot profiles showed different patterns of exploration and learning dynamics depending on the perceptual complexity of the environment. Our results also showed that the two profiles exhibit different behavioral dynamics as a function of their different processing of the comfort provided by the caregiver. The exploration dynamics of both robots produced a different learning “experiences” for the two robot profiles. The “needy” profile stopped more often and spent more time learning than the “independent” one. The “independent” profile showed longer exploration episodes following the relief due to the comfort provided lowers the level of arousal for a longer time.

The results from these two sets of experiments show that our architecture can allow a human interacting with the robot to influence and even decide on the granularity of the exploration. Adapted to the difficulty of the learning task at hand, the amount of comfort provided by the human can lower a high level of arousal to a medium level, causing the robot to focus on the stimulus it is currently attending to. If even more comfort is provided, the level of arousal will drop below the low threshold, triggering an exploratory behavior that drives the robot to move away from the stimulus it was attending to. Our results also show that different types of interaction and “caregiving styles” affect differentially the regulatory, exploratory and learning patterns of the two robot profiles. The interaction dynamics between the immediate responsiveness of the caregiver and each profile gave rise to a responsive and constantly present caregiver in the case of the “needy” robot, and to a responsive but more “hands off” caregiver in the case of the “independent” robot. The profiles of the robots and the caregiving styles matched to give rise to different but equally valid regulatory, exploratory and learning patterns.

Taking a developmental approach, this robot architecture and its close interrelation with the behavior of and interaction with a human “caregiver” can provide a basis for the *personalization* and *adaptation* of the behavior of the robot to the interaction profile of the human, based on the features of the environment or on the specific contexts in which the caregiver interacted the most with the robot. Through his/her interventions, the human can decide when closer attention has to be paid to specific aspects of the environments and when to discard the current perceptual context, biasing the learning of the robot in a way that meets his/her preferences or needs.

In addition to the comparison of the two stereotypically designed “idealized” profiles, we modified the architecture to make the affective regulatory behavior of the robot *adaptive* to the *responsiveness* of the human. This component was inspired by the literature on parental caring style and the dimensions used to assess it (De Wolf and van Ijzendoorn, 1997). The notion of *responsiveness* has been linked to a carer's ability to respond to an infant's demands in a timely and accurate manner. The hypotheses on the formation of patterns of attachment (Ainsworth et al., 1978) postulate that infants adapt to the interactive style of their caregiver and their “trust” in the caregiver's ability to soothe them biases their behavior. In a similar manner, we introduced this adaptive element in the architecture to provide the robot with a tool to cope with real-time variations in the caregiver's availability to respond to regulatory behaviors. The architecture modulates the effect of the comfort provided by the human by modifying the parameters used to process the comfort provided. The robot can therefore in turn modify its own profile autonomously along the “needy” and “independent” dimension. The more comfort is provided to the robot, the more the robot leans toward the “needy” profile. When requests are not responded to, the behavior of the robot moves toward a more “independent” profile. In a real-world scenario, this adaptivity should help a robot tune the quantity and frequency of its affective regulatory behavior to the behavior of the human it interacts with.

In the near future, we plan to assess the adaptive component based on the *responsiveness* of the human with naive users, in particular (diabetic) children between 8 and 12 years of age, which is the target population of the ALIZ-E project, to which this study contributes.

## Funding

This research was funded by the European Commission as part of the ALIZ-E project (FP7-ICT-248116). The opinions expressed are solely the authors'.

### Conflict of interest statement

The authors declare that the research was conducted in the absence of any commercial or financial relationships that could be construed as a potential conflict of interest.
